# Retinal Vasculitis as a Rare Presentation of Microscopic Polyangiitis

**DOI:** 10.7759/cureus.76532

**Published:** 2024-12-28

**Authors:** Cristiane P Macedo, Andreia M Matos, João Chaves, Patricia A Mendes

**Affiliations:** 1 Internal Medicine, Unidade Local de Saúde de Coimbra, Coimbra, PRT; 2 Ophthalmology, Unidade Local de Saúde de Coimbra, Coimbra, PRT

**Keywords:** anca-associated vasculitis, eosinophilic granulomatosis with polyangiitis, granulomatosis with polyangiitis, microscopic polyangiitis, ocular manifestations, retinal vasculitis

## Abstract

Microscopic polyangiitis (MPA) is a rare, autoimmune, small-vessel vasculitis usually described with the presence of perinuclear antineutrophil cytoplasmic antibodies (p-ANCA). It encompasses a broad spectrum of clinical features, including fatigue, weight loss, fever, arthralgia, skin lesions, and involvement of the lungs or kidneys. Ocular manifestations, however, are extremely rare. We report a case of a 60-year-old Caucasian male who presented with sudden left-hand weakness, fever, and *amaurosis fugax*. He also complained of fatigue and stocking-glove paresthesia over the preceding two months. Neurological evaluation excluded acute stroke, and ophthalmologic examination, supported by fluorescein angiography, revealed mild right-sided temporal retinal vasculitis without other retinal changes. Laboratory investigations demonstrated inflammatory anemia, elevated erythrocyte sedimentation rate, mild proteinuria, autoimmune hypothyroidism, and positive p-ANCA-myeloperoxidase (MPO) with a nuclear-dense anti-nuclear antibodies (ANA) pattern. Renal biopsy confirmed crescentic vasculitis, while nerve biopsy indicated small-vessel vasculitis. Treatment with prednisolone and azathioprine resulted in clinical improvement. This case highlights retinal vasculitis as a rare manifestation of MPA.

## Introduction

Microscopic polyangiitis (MPA) is a rare (1:100,000 people), autoimmune, systemic small-vessel vasculitis. It is one of the antineutrophil cytoplasmic antibody (ANCA)-associated vasculitis, characterized by pauci-immune necrotizing vasculitis and strong association with perinuclear ANCA (p-ANCA) targeting myeloperoxidase (MPO) [1,2].

The clinical presentation of MPA is highly variable, with symptoms often depending on the organs involved. This condition usually presents with a wide spectrum of clinical features: fatigue, weight loss, fever, and arthralgia [3,4]. The kidneys and lungs are most frequently affected, presenting with manifestations such as rapidly progressive glomerulonephritis and pulmonary capillaritis, respectively [5]. Neurological and musculoskeletal symptoms are also common, contributing to a broad spectrum of systemic involvement. This vasculitis can also be present in a single organ [6].

Ocular manifestations are highly uncommon at diagnosis and described only in 4.1% of patients with MPA [7]. Among ocular manifestations, retinal vasculitis is one of the rarest, presenting in only 2.7% of MPA patients [8]. It poses a unique diagnostic challenge as its clinical signs are subtle, often requiring advanced imaging techniques such as fluorescein angiography for confirmation. Ocular involvement in systemic vasculitis, including MPA, is a critical area of study because it may serve as an early indicator of systemic disease or relapse, underscoring the importance of timely diagnosis and treatment [9].

Existing literature on retinal vasculitis in MPA is sparse, with most reports highlighting isolated or atypical presentations. However, the underlying pathophysiological mechanisms, predictors of ocular complications, and optimal treatment strategies remain incompletely understood [8]. We present a case of retinal vasculitis as a presentation of MPA.

## Case presentation

**Figure 1:**
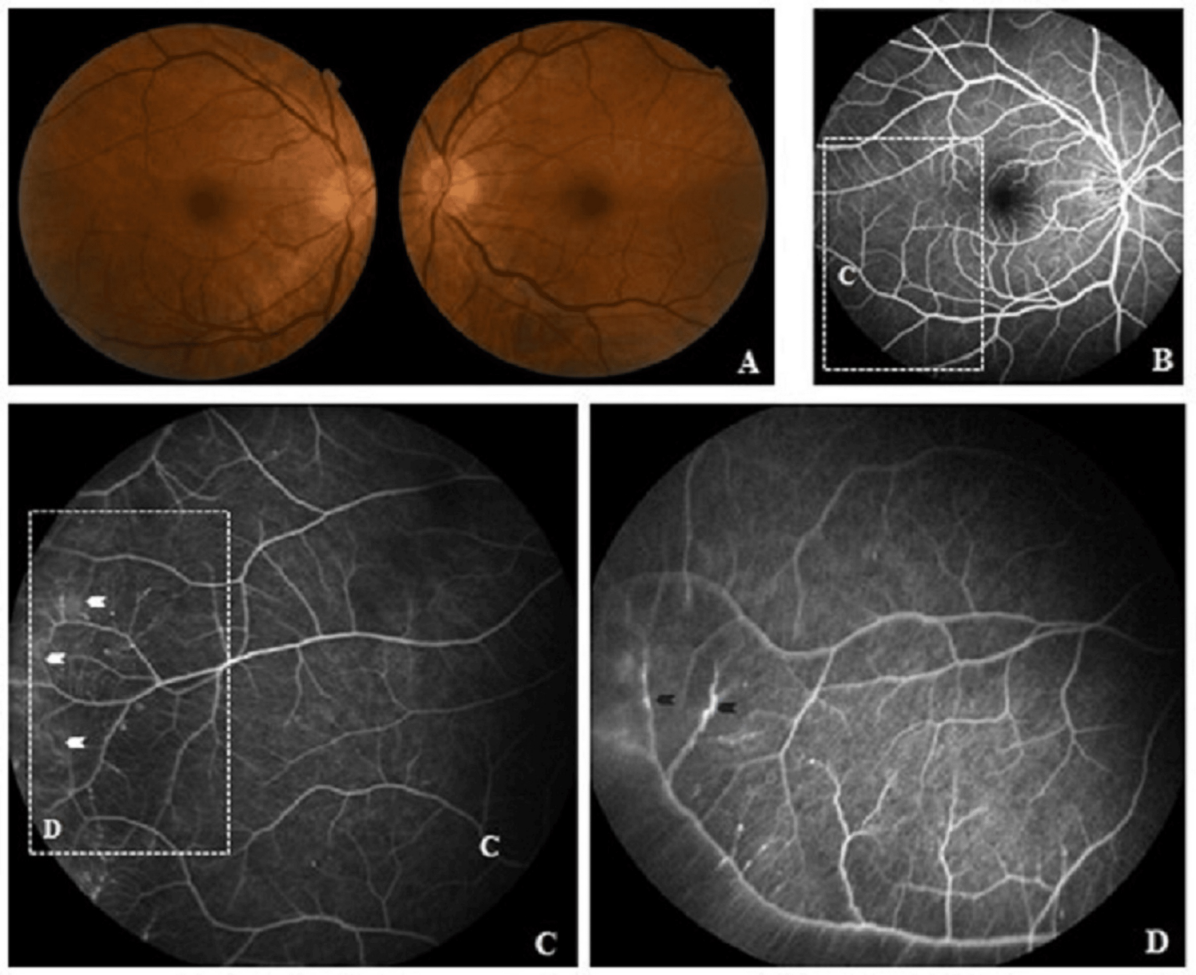
Fundoscopy and fluorescein angiography of retinal vasculitis (A) Normal bilateral fundus photograph with (B) fluorescein angiography disclosing mild, right-side retinal vasculitis on image magnification in (C). The vessels of the temporal periphery arcade show inflammation denoted by leakage of the vessel wall (white arrows) and (D) retinal vascular sheathing (blue arrows). Macular edema and optic nerve head inflammation were absent in (A) and (B).

A Caucasian 60-year-old male was admitted to an internal medicine ward after a sudden loss of strength in the left hand, a fever, and *amaurosis fugax *of the right eye. For the past two months, he complained of fatigue, weight loss, cold intolerance, proximal muscular pain, and progressive asymmetrical stocking-glove pattern paresthesia. The patient denied ear or throat symptoms, ocular pain, red eye, diplopia, photophobia, mouth or genital ulcers, alopecia, sinusitis, arterial thrombosis, or past relapsing polychondritis. His past history consisted of alcohol (80 grams/day) and tobacco abuse (eight pack years), dyslipidemia, essential hypertension, obesity, and previous myocardial infarction. He denied respiratory symptoms, and his pulmonary function tests were normal. He exhibited painless, bilateral, palpable purpura on the legs with no alterations upon objective examination of the nails, chest, and abdomen. A neurological exam revealed radial nerve dysfunction without other deficits or acute stroke in angiography. The ophthalmologic exam showed normal bilateral visual acuity (20/20 on *Snellen* chart), without scleritis, keratitis, or uveitis, but disclosing mild, right-side, temporal retinal vasculitis in fluorescein angiogram (Figure 1), in the absence of vessels occlusion, hemorrhages, exudates, or retinal detachment. Macular edema and optic nerve head inflammation were absent, with no relative afferent pupillary defect.

Blood tests showed inflammatory anemia, transient eosinophilia <10%, an erythrocyte sedimentation rate of 50 mm/h, mild proteinuria without casts, and autoimmune hypothyroidism. There was strong positivity for p-ANCA (anti-MPO positive) with a nuclear dense fine speckled ANA pattern (1:640). Liver enzymes, creatinine, ionogram, protein electrophoresis, immunoglobulins, c-ANCA, anti-CCP, ds-DNA, cryoglobulins, and myositis antibodies were normal or negative. An electromyogram confirmed motor sensory axonal polyneuropathy. Renal biopsy showed crescentic glomerulonephritis and nerve biopsy was compatible with severe chronic axonal lesion with CD3+ small-vessel vasculitis, without granulomas or immunoglobulin deposits. Additional blood, cerebrospinal fluid (CSF), and imaging tests ruled out infection or neoplasm: serological analysis for acute infection for cytomegalovirus, Epstein-Barr, Borrelia, syphilis, Toxoplasma, chlamydia, herpes simplex, HIV 1-2, and A, B, and C hepatitis viruses were negative. Blood, urine, and CSF cultures for mycobacteria were negative, and biochemical, cytological, and microbiological analyses of the CSF were also normal. Computed tomography (CT) angiography, brain and cervical magnetic resonance imaging (MRI) did not show vascular lesions or neoplasm, and chest and abdominal CT, endoscopy, and colonoscopy were normal. Positron emission tomography-fludeoxyglucose F18 (PET-FDG18) did not show additional disease activity. After high clinical suspicion and excluding acute infection (skin and nerve biopsy results were still pending), induction therapy was started with 1 mg/kg/day of prednisolone in monotherapy. One month later, due to the maintenance of mild proteinuria, a renal biopsy was performed showing crescentic glomerulonephritis. At this time, 150 mg a day of azathioprine was added to the previous therapy. After three months of treatment, clinical recovery, and p-ANCA normalization were noticed. The patient initiated progressive prednisolone reduction. After three years, he is stable with prednisolone 5 mg/day and azathioprine 75 mg/day. 

## Discussion

We report an uncommon finding in a rare autoimmune condition: a case of retinal vasculitis associated with MPA. The revised 2012 Chapel Hill Consensus 8 defines MPA as a non-granulomatous and necrotizing small- and medium-sized vessel vasculitis with few or no immune deposits. Our case fulfills these criteria, supported by histological findings of necrotizing small-vessel vasculitis in the absence of granulomas, in a sexagenarian Caucasian male with weight loss, myalgia, mononeuropathy, and strong positivity for p-ANCA-MPO. The clinical expression of MPA is variable, with multisystem involvement that can include necrotizing glomerulonephritis, constitutional symptoms such as weight loss and fever, paranasal sinus involvement, myalgias, arthralgias, lung involvement, mononeuritis multiplex, and cutaneous lesions predominantly affecting the lower extremities [6].

Inflammatory ocular disease occurs in approximately one-fifth of patients with ANCA-associated vasculitis. However, ophthalmologic manifestations were significantly more frequent in granulomatosis with polyangiitis (34.1%) than in eosinophilic granulomatosis with polyangiitis (11.1%), polyarteritis nodosa (10.7%), or MPA (8.9%) as in our case [10].

A recent large-scale study from multiple Chinese medical centers, the largest Asian cohort of patients with ANCA-associated vasculitis to date, reported that only 7.37% of patients with MPA presented with eye or orbital manifestations, retinal vasculitis presenting in just 2.76% of total cases. They also identified analytical predictors for eye and orbital involvement, including retinal vasculitis. In their cohort, elevated eosinophil percentage, high-sensitivity C-reactive protein (hsCRP), and a lower CD4 + T cell/CD8 + T cell ratio (T4/T8) were associated with retinal involvement. These findings emphasize the potential for systemic inflammation markers to predict specific ocular manifestations [8].

Our case is consistent with some of these findings, as our patient exhibited elevated eosinophils and systemic markers of inflammation. By presenting detailed laboratory findings, including positive p-ANCA and autoimmune thyroid markers, our report contributes to the growing body of evidence supporting systemic inflammation as a predictor for ocular manifestations.

Our case report provides detailed analytical data, summarized in Table 1, which can offer further insights into possible predictors of retinal vasculitis in MPA. Incorporating such findings into clinical practice may facilitate earlier identification and treatment of at-risk patients and contribute to the growing body of evidence supporting systemic inflammation as a predictor for ocular manifestations.

**Table 1:** Analytical study ANA: anti-nuclear antibodies; anti-MPO antibodies: anti-myeloperoxidase antibodies; anti-TPO antibodies: anti-thyroid peroxidase antibodies; p-ANCA: perinuclear anti-neutrophil cytoplasmic antibodies; TSH: thyroid stimulating hormone; T4: thyroxine

Parameter	Result	Reference range
Hemoglobin	12.5 g/dL	13.5-16.5
Eosinophils	0.83x10^9 ^/L	0.02-0.5
Erythrocyte sedimentation rate	50 mm/h	1-20
Ferritin	1044.6 ng/mL	30-300
C-reactive protein	10.87mg/dL	<0.5
Urinary proteins	406 mg/time	50-80
TSH	8.9 uUI/mL	0.4-4
T4	0.4 ng/dL	0.7-1.5
ANA	1:640	
p-ANCA	Positive	
Anti-TPO antibodies	83 UI/mL	<5.6
Anti-MPO antibodies	35 IU/mL (strong positive)	<5

In the context of MPA, ocular involvement appears more common in association with c-ANCA-PR3 (odds ratio (OR) 2.3), while p-ANCA-MPO vasculitis is linked to an increased risk of renal disease (OR 2.6) and alveolar hemorrhage (OR 2) [4]. All parts of the eye and orbit can be affected, but anterior segment inflammation is more common than posterior segment involvement [4,6,11]. In this matter, Rothschild et al. reported retinal vasculitis in only 1% (three cases) of a total of 280 MPA patients, 25 of those with ocular manifestations [10]. Other series of MPA patients described ocular involvement in 1.2% to 4% of cases [4,6,8,11], but concerns about potential subclinical microvascular abnormalities were not addressed [6,10].

Major ocular presenting signals were red eyes in 56% of cases, primarily due to conjunctivitis or episcleritis, and blurred vision in 16%. While less common, sudden visual loss, oculomotor nerve palsy, and optic neuropathy may also occur [6,10]. Retinal vasculitis has been documented in infectious diseases such as toxocariasis, toxoplasmosis, tuberculosis, bartonellosis, Q fever, Lyme disease, and syphilis, as well as in inflammatory conditions like Behçet’s disease, systemic lupus erythematosus, rheumatoid arthritis, sarcoidosis, relapsing polychondritis, and inflammatory bowel disease. Despite this wide range of associations, retinal vasculitis remains an exceedingly rare manifestation of MPA, underscoring the importance of its recognition in clinical practice [3,8,10,12].

## Conclusions

This case underscores retinal vasculitis as an extremely rare manifestation of MPA, a systemic vasculitis characterized by multisystem involvement and predominantly associated with p-ANCA. While ocular manifestations of MPA are uncommon, their recognition is essential, as they may represent early or atypical presentations of the disease. *Amaurosis fugax*, polyneuropathy with progressive asymmetrical stocking-glove pattern paresthesia, fatigue, weight loss, and sudden loss of strength are clinical findings that may suggest systemic vasculitis. In this scenario, we have to search for organ- or life-threatening diseases to decide the best treatment options. Retinal vasculitis confirmed by fluorescein angiography and biopsy-proven small-vessel vasculitis without granulomas aligns with the revised 2012 Chapel Hill Consensus Criteria for MPA. Laboratory findings such as elevated eosinophil percentage and high-sensitivity C-reactive protein may be related to retinal involvement. In our case, effective management first with corticosteroids and then azathioprine resulted in clinical improvement and p-ANCA normalization.

This case emphasizes the need to maintain a high level of suspicion for rare ocular manifestations in patients with systemic vasculitis, as early diagnosis and appropriate treatment can prevent further complications. Further studies and case reports are warranted to better understand the frequency and clinical significance of ocular involvement in MPA.

This case report not only underscores the rarity of retinal vasculitis in MPA but also provides analytical data that could contribute to identifying new predictive markers for ocular involvement in systemic vasculitis, complementing recent findings from larger cohorts.
